# Tobacco use, exposure to secondhand smoke, and cessation counseling among medical students: cross-country data from the Global Health Professions Student Survey (GHPSS), 2005-2008

**DOI:** 10.1186/1471-2458-11-72

**Published:** 2011-02-01

**Authors:** Charles W Warren, Dhirendra N Sinha, Juliette Lee, Veronica Lea, Nathan R Jones

**Affiliations:** 1 Office on Smoking and Health, US Centers for Disease Control and Prevention, Atlanta, GA, USA; 2 Tobacco Free Initiative, South East Asia Region, World Health Organization, Delhi, India; 3 Paul P. Carbone Comprehensive Cancer Center, University of Wisconsin, Madison, WI, USA

## Abstract

**Background:**

GHPSS is a school-based survey that collects self-administered data from students in regular classroom settings. GHPSS produces representative data at the national or city level in each country. This study aims to investigate the prevalence of tobacco use, exposure to secondhand smoke, and cessation counseling among medical students using the GHPSS data.

**Methods:**

The Global Health Professions Student Survey (GHPSS) was conducted among 3^rd ^year medical students in 47 countries and the Gaza Strip/West Bank from 2005-2008 to determine the prevalence of tobacco use and amount of formal training in cessation counseling.

**Results:**

In 26 of the 48 sites, over 20% of the students currently smoked cigarettes, with males having higher rates than females in 37 sites. Over 70% of students reported having been exposed to secondhand smoke in public places in 29 of 48 sites. The majority of students recognized that they are role models in society (over 80% in 42 of 48 sites), believed they should receive training on counseling patients to quit using tobacco (over 80% in 41 of 48 sites), but few reported receiving formal training (less than 40% in 46 of 48 sites).

**Conclusion:**

Tobacco control efforts must discourage tobacco use among health professionals, promote smoke free workplaces, and implement programs that train medical students in effective cessation-counseling techniques.

## Background

"Tobacco use presents a rare confluence of circumstances: 1) a highly significant health threat; 2) a disinclination among clinicians to intervene consistently; and 3) the presence of effective interventions," a recent publication concluded [1]. The World Health Organization (WHO) estimates globally over 1 billion people currently smoke tobacco; with approximately 5 million deaths a year attributed to tobacco [2]. If current trends continue, WHO estimates tobacco attributable mortality will exceed 8 million per year by 2030. A disproportionate share of the global tobacco burden falls on developing countries, where 84% of 1.3 billion current smokers reside.

A number of studies have found that smoking cessation rates increase after even brief or simple counseling by physicians [3-6]. These studies have shown that physicians are willing to provide counseling and patients who smoke are receptive to counseling but in many cases the counseling is not repeated at subsequent visits nor is follow-up done. In addition, barriers have been identified that reduce the willingness of physicians to provide patient counseling, including time constraints during the consultation, physicians' lack of confidence in their ability to provide effective advice, and the smoking status of the physician.

In the WHO Framework Convention on Tobacco Control (WHO FCTC), the first public health treaty developed to counteract the global tobacco epidemic by encouraging countries to implement provisions to reduce the burden of tobacco, Article 12 calls on parties to implement effective and appropriate training on tobacco control to health workers including health professionals [7]. The 2008 WHO "MPOWER Report", a package of six tobacco policies proven to reduce the global tobacco epidemic, notes that "managing tobacco dependence is primarily the responsibility of a country's health-care system, including government, social security, NGOs and private clinical services"[2]. The Report identifies two primary interventions that will likely enhance quitting among tobacco users: 1) counseling by physicians and other health care workers during regular medical care (and quit line and community programs); and 2) access to low-cost pharmacological therapy [2].

Only a few studies have collected information on tobacco use, exposure to secondhand smoke (SHS), and training to provide cessation counseling among medical students. These studies used different sampling methods, questionnaires, and data collection procedures, and very few are from low or middle-income countries [8]. WHO, U.S. Centers for Disease Control and Prevention, and the Canadian Public Health Association have attempted to overcome these limitations by developing and implementing the Global Health Professions Student Survey (GHPSS) [9]. The data reported in this study come from GHPSS conducted among 3^rd ^year medical students in 47 countries and the Gaza Strip/West Bank (identified as "sites" for the remainder of this paper).

## Methods

The Medical GHPSS is part of the Global Tobacco Surveillance System, which collects data through four surveys: the Global Youth Tobacco Survey, the Global School Personnel Survey, the Global Adult Tobacco Survey, and GHPSS. GHPSS is a school-based survey of 3rd year students pursuing advanced degrees in dentistry, medicine, nursing or pharmacy. GHPSS has a standardized methodology for selecting schools, uniform data processing procedures and uses a core questionnaire [9].

The Medical GHPSS used two sample designs depending upon the total enrollment of 3^rd ^year students and the number of schools. In sites where the number of medical students exceeded 1000 and the number of schools was greater than 10, a representative sample of schools was selected probability proportionate to size, and all 3^rd ^year medical students in the schools were included. Sites with a sample of medical students included: Egypt, Bolivia, Mexico, Peru, Bangladesh, India, Indonesia, Nepal, and South Korea. For all other sites a census of schools and students was conducted where all schools and all 3rd year medical students were included in the survey (Table 1).

**Table 1:** Response Rates by Region and Country, Medical Global Health Professions Student Survey, 2005-2008

Country (Site)	Year	School Response Rate (%)	Class Response Rate (%)	Student Response Rate (%)	Overall Response Rate (%)	Number of 3rd Year Students
**AFRICAN REGION (AFR)**						

Algeria	2007	100.0	100.0	78.0	78.0	1,088

Kenya	2008	100.0	100.0	73.0	73.0	227

Niger	2008	80.0	100.0	77.7	62.1	754

Uganda	2005	100.0	100.0	85.3	85.3	154

**EASTERN MEDITERRANEAN REGION (EMR)**						

Egypt*	2005	100.0	100.0	93.9	93.9	1,770

Gaza Strip/West Bank	2007	100.0	100.0	92.3	92.3	81

Iran	2007	90.2	100.0	50.8	45.9	1,065

Iraq	2005	100.0	100.0	81.3	81.3	247

Lebanon	2006	100.0	100.0	70.0	70.0	254

Libyan Arab Jamahiriya	2006	90.0	100.0	77.9	70.1	1,488

Saudi Arabia	2006	100.0	100.0	62.6	62.6	481

Sudan	2007	100.0	100.0	64.5	64.5	591

Syrian Arab Republic	2006	100.0	100.0	90.7	90.7	1,170

Tunisia	2007	100.0	100.0	68.2	68.2	686

**EUROPEAN REGION (EUR)**						

Albania	2005	100.0	100.0	93.2	93.2	136

Armenia	2006	100.0	100.0	90.0	90.0	177

Bosnia & Herzegovina	2006	100.0	100.0	94.4	94.4	421

Croatia	2005	100.0	100.0	98.5	98.5	395

Czech Republic	2006	100.0	100.0	94.7	94.7	690

Kyrgyzstan	2008	100.0	100.0	92.7	92.7	112

Lithuania	2006	100.0	100.0	64.6	64.6	221

Russian Federation	2006	77.8	100.0	100.0	77.8	1,797

Serbia	2006	100.0	100.0	81.6	81.6	775

Slovakia	2006	100.0	100.0	91.9	91.9	378

Slovenia	2007	100.0	100.0	69.9	69.9	142

**REGION OF THE AMERICAS (AMR)**						

Argentina	2005	100.0	100.0	85.9	85.9	306

Bolivia*	2006	100.0	100.0	97.1	97.1	2,380

Brazil (Rio de Janeiro)	2006	100.0	100.0	78.5	78.5	761

Chile	2008	95.7	100.0	82.8	79.2	1,075

Costa Rica	2006	100.0	100.0	100.0	100.0	279

Cuba (Havana)	2008	100.0	100.0	80.2	80.2	477

Guatemala	2008	100.0	100.0	72.3	72.3	351

Jamaica	2008	100.0	100.0	65.5	65.5	107

Mexico*	2006	93.3	100.0	83.4	77.8	2,226

Panama	2008	100.0	100.0	85.7	85.7	285

Paraguay	2008	100.0	100.0	89.1	89.1	396

Peru*	2006	94.4	100.0	97.4	92.0	1,373

Uruguay	2008	100.0	100.0	98.1	98.1	346

**SOUTH-EAST ASIA REGION (SEAR)**						

Bangladesh*	2006	100.0	100.0	77.0	77.0	777

India*	2005	86.7	100.0	89.1	77.2	1,176

Indonesia*	2006	100.0	100.0	77.4	77.4	1,580

Myanmar	2006	100.0	100.0	87.5	87.5	1,430

Nepal*	2005	83.3	100.0	74.9	62.5	327

Sri Lanka	2006	100.0	100.0	78.0	78.0	617

Thailand	2006	100.0	100.0	63.8	63.8	781

**WESTERN PACIFIC REGION (WPR)**						

Cambodia	2005	100.0	100.0	65.3	65.3	111

South Korea*	2006	82.4	100.0	53.3	43.9	741

Viet Nam	2007	100.0	100.0	88.9	88.9	1,423

* sample of medical schools probability proportional to size

All sites were nominated by their Ministries' of Health in conjunction with their respective WHO regional offices. The majority of the 48 sites included in this study were low to middle income countries. The Medical GHPSS was conducted in schools during regular lectures and class sessions. Anonymous, self-administered data collection procedures were used. For all sites that conducted a census, a finite population correction factor was applied to take into account non-response and used in the variance of the estimates. For sites that did not conduct a census, a weighting factor was applied to each student record to adjust for nonresponse and variation in the probability of selection. SUDAAN was used to calculate weighted prevalence estimates and standard errors (SE) of the estimates (95% confidence intervals (CI) were calculated from the SEs) [10]. T-tests were used to determine differences between subpopulations [11,12]. In this paper, differences in proportions are considered statistically significant if the t-test p value was less than 0.05. Results in this report are presented by WHO region with select countries highlighted. The six WHO regions are the African Region (AFR), the Eastern Mediterranean Region (EMR), the European Region (EUR), the Americas Region (AMR), the South East Asian Region (SEAR), and the Western Pacific Region (WPR). Data were not aggregated at the WHO regional level as the 47 countries represent only a fraction of the total Member States in each region: AFR 4 of 46; AMR 13 of 35; EMR 9 of 12; EUR 11 of 53; SEAR 7 of 11; and WPR 3 of 27. Data presented here are an expansion on previous published data [9].

For sites conducting the Medical GHPSS, the school response rate was 100% in 38 of the 48 sites; the classroom response rate was 100% in all sites; the student response rate ranged from 50.8% to 100%; and the overall response rate ranged from 43.9% to 100% (Table 1). The number of students who participated in each survey varied due to the number of schools and students in each sample design.

This report includes information on current cigarette smoking, defined as those who answered one or more days to the question, "During the past 30 days (one month), on how many days did you smoke cigarettes?", current use of tobacco products other than cigarettes, defined as those who answered one or more days to the question, "During the past 30 days (one month), on how many days did you use chewing tobacco, snuff, bidis, cigars, or pipes (adapted to fit each country)?", exposure to SHS at home and in public places, official school policies banning smoking in school buildings and clinics, and policy enforcement. Additionally, attitude questions were asked regarding: health professionals as role models for their patients, whether health professionals think they should get training in patient cessation techniques, and if they have ever received formal training on such cessation counseling techniques. The final country questionnaires were translated into local languages as needed and back-translated to check for accuracy. Cognitive lab testing of the questions was performed by an out-sourced independent survey company (RTI International).

### Ethical Considerations

This study was a collaboration between the WHO region offices and CDC and the protocol was approved by both organizations. At CDC and WHO region offices, GHPSS was approved following ethical review procedures for surveillance systems. Detailed information about the study was provided verbally to the potential participants and informed consent was obtained from participating students. Data collection procedures assured confidentiality by the use of self-administered, anonymous questionnaires. Ethical approval from each of the participating universities was not required as the study was voluntary and confidentially was fully guaranteed.

## Results

### Student Characteristics

The percentage of medical students who were females was over 60% in 23 of the 48 sites (including all 11 sites in EUR); the percent female was less than 40% in 6 sites (Table 2). Over 90% of the students were less than age 25 in every site except Niger, Uganda, Lebanon, Bosnia & Herzegovina, Argentina, Bolivia, Brazil, Costa Rica, Guatemala, Jamaica, Paraguay, and South Korea.

**Table 2:** Prevalence of Current Cigarette Smoking by Region and Country, Medical Global Health Professions Student Survey, 2005-2008 & MPOWER 2008 Adult Data

	Current Cigarette Smoking			
	
	GHPSS Medical Students		MPOWER 2008 Adult	
**Country** **(Site)**	**Male %** **(95% CI)**	**Female %** **(95% CI)**	**Male %** **(95% CI)**	**Female %** **(95% CI)**

**AFRICAN REGION (AFR)**				

Algeria	23.7 (21.3 - 26.2)	3.0 (2.5 - 3.7)	28.7 (26.3 - 31.2)	0.2 (0 - 0.4)

Kenya	10.9 (8.0 - 14.8)	8.7 (5.9 - 12.6)	22.2 (19.4 - 24.9)	0.9 (0.5 - 1.3)

Niger	42.2 (37.9 - 46.7)	15.0 (9.6 - 22.8)	NA	NA

Uganda	4.1 (2.7 - 6.3)	0	15.7 (13.6 - 17.8)	1.2 (0.8 - 1.5)

**EASTERN MEDITERRANEAN REGION (EMR)**				

Egypt	12.9 (9.9 - 16.5)	1.2 (0.5 - 3.0)	22.7 (20.7 - 24.7)	3.5 (2.5 - 4.5)

Gaza Strip/West Bank	39.1 (33.8 - 44.6)	9.4 (6.8 - 12.8)	NA	NA

Iran	11.6 (9.0 - 14.9)	2.8 (1.9 - 4.0)	21.4 (17.5 - 25.4)	1.7 (1.1 - 2.2)

Iraq	20.4 (17.2 - 24.1)	15.1 (12.5 - 18.2)	25.2 (21.1 - 29.3)	1.3 (0.4 - 2.1)

Lebanon	36.1 (31.8 - 40.7)	18 (14.4 - 22.3)	29 (24.2 - 33.9)	6.9 (2.7 - 11.2)

Libyan Arab Jamahiriya	22.3 (20.1 - 24.6)	1.9 (1.4 - 2.7)	NA	NA

Saudi Arabia	13.1 (10.0 - 16.9)	9.6 (6.2 - 14.8)	25.2 (21.4 - 28.9)	3.0 (1.2 - 4.8)

Sudan	19.6 (15.9 - 23.9)	1.3 (0.8 - 2.3)	NA	NA

Syrian Arab Republic	24.3 (23.3 - 25.3)	4.8 (4.2 - 5.5)	41.2 (16.5 - 65.9)	NA

Tunisia	26.5 (22.7 - 30.7)	4.1 (3.2 - 5.4)	47.4 (44.7 - 50.1)	1.0 (0.6 - 1.4)

**EUROPEAN REGION (EUR)**				

Albania	65.1 (59.8 - 69.9)	35.7 (32.8 - 38.7)	39.6 (26.6 - 52.7)	3.9 (0.6 - 7.2)

Armenia	52.5 (41.7 - 63.0)	8.3 (5.2 - 13.1)	52.9 (45.2 - 60.5)	4.0 (1.5 - 6.5)

Bosnia & Herzegovina	45.0 (43.0 - 47.0)	37.8 (36.4 - 39.2)	48.8 (42.3 - 55.3)	32.0 (26.3 - 37.8)

Croatia	35.9 (31.5 - 40.4)	37.1 (34.1 - 40.3)	37.5 (35.7 - 39.3)	25.4 (24.3 - 26.5)

Czech Republic	26.2 (21.9 - 30.9)	19.8 (17.2 - 22.5)	35.9 (29.4 - 42.5)	23.4 (16.4 - 30.3)

Kyrgyzstan	50.0 (45.5 - 54.5)	27.9 (24.8 - 31.3)	45 (36.9 - 53.2)	2.2 (1.3 - 3.0)

Lithuania	48.6 (39.7 - 57.7)	19.5 (15.8 - 23.8)	44.4 (37.3 - 51.5)	17.6 (15.0 - 20.2)

Russian Federation	46.1 (44.2 - 48.1)	34.9 (33.5 - 36.2)	70.2 (59.2 - 81.3)	23.2 (16.7 - 29.7)

Serbia	31.2 (28.7 - 33.8)	36.7 (34.9 - 38.6)	41.4 (36.5 - 46.3)	40.4 (35.7 - 45.1)

Slovakia	36.5 (33.6 - 39.4)	27.9 (26.2 - 29.6)	41.4 (34.4 - 48.4)	18.5 (13.2 - 23.8)

Slovenia	22.5 (15.9 - 30.8)	20.2 (16.0 - 25.1)	29.6 (23.6 - 35.5)	19.9 (15.1 - 24.7)

**REGION OF THE AMERICAS (AMR)**				

Argentina	33.4 (30.4 - 36.4)	36.5 (34.1 - 39.1)	34.3 (30.8 - 37.8)	22.7 (20.0 - 25.4)

Bolivia	50.3 (43.9 - 56.7)	31.7 (24.9 - 39.4)	35.7 (27.8 - 43.6)	27.3 (24.0 - 30.6)

Brazil (Rio de Janeiro)	19.5 (17.5 - 21.6)	14.6 (13.1 - 16.3)	NA	NA

Chile	27.1 (25.5 - 28.9)	29.3 (27.5 - 31.3)	42.2 (33.7 - 50.7)	30.1 (25.2 - 35.0)

Costa Rica	38.6	29.3	26.7 (22.5 - 30.9)	7.3 (5.8 - 8.9)

Cuba (Havana)	41.1 (38.0 - 44.3)	21.2 (19.1 - 23.5)	37.0 (22.4 - 51.6)	27.3 (20.8 - 33.8)

Guatemala	27.5 (23.4 - 32.1)	17.8 (14.6 - 21.6)	24.8 (20.8 - 28.9)	3.9 (3.0 - 4.8)

Jamaica	12.5 (7.1 - 21.2)	4.2 (2.1 - 8.0)	17.7 (9.9 - 25.5)	7.5 (5.0 - 9.9)

Mexico	41.3 (36.0 - 46.8)	30.8 (24.7 - 37.6)	37.6 (30.2 - 45.0)	12.4 (8.9 - 15.9)

Panama	11.2 (8.7 - 14.1)	11.1 (9.2 - 13.5)	NA	NA

Paraguay	25.3 (23.2 - 27.7)	19.9 (18.1 - 21.8)	33.2 (29.4 - 37.0)	14.4 (12.2 - 16.5)

Peru	43.5 (36.2 - 51.1)	22.2 (16.7 - 28.8)	NA	NA

Uruguay	31.3 (29.5 - 33.2)	32.4 (31.2 - 33.7)	36.6 (32.2 - 41.0)	25.8 (22.1 - 29.6)

**SOUTH-EAST ASIA REGION (SEAR)**				

Bangladesh	46.5 (37.6 - 55.6)	4.4 (1.2 - 14.1)	41.0 (33.2 - 48.8)	0.7 (0.3 - 1.1)

India	17.7 (14.5 - 21.3)	2 (0.9 - 4.2)	25.8 (20.8 - 30.8)	0.6 (0.4 - 0.8)

Indonesia	19.8 (12.5 - 29.7)	2.3 (1.2 - 4.5)	61.8 (54.3 - 69.3)	3.7 (3.3 - 4.2)

Myanmar	24.7 (23.5 - 26.1)	1.2 (0.9 - 1.6)	42.5 (37.0 - 47.9)	10.1 (9.0 - 11.2)

Nepal	34.5 (13.1 - 64.7)	4.0 (1.2 - 12.5)	25.2 (20.4 - 30.0)	22.4 (15.3 - 29.5)

Sri Lanka	8.6 (7.0 - 10.7)	1.1 (0.7 - 1.8)	24.4 (19.6 - 29.1)	0.4 (0.2 - 0.7)

Thailand	1.0 (0.5 - 2.1)	3.0 (2.1 - 4.3)	37.3 (32.9 - 41.8)	3.0 (2.9 - 3.1)

**WESTERN PACIFIC REGION (WPR)**				

Cambodia	9.2 (6.0 - 13.9)	0.0	30.3 (26.5 - 34.1)	10.8 (10.0 - 11.6)

South Korea	22.5 (18.1 - 27.5)	3.5 (1.6 - 7.2)	53.8 (51.4 - 56.1)	5.6 (4.9 - 6.4)

Viet Nam	20.8 (19.7 - 21.9)	2.6 (2.3 - 3.0)	42.0 (36.4 - 47.6)	1.9 (1.2 - 2.5)

NA Data not available

### Tobacco Use

Among medical students, 3 sites had current smoking rates above 40% (Albania, Bosnia & Herzegovina, and Bolivia) and 3 sites had rates less than 5% (Uganda, Sri Lanka, and Thailand) (Table 3). Males were more likely than females to smoke cigarettes in 37 of 48 sites; females had higher rates than males in Serbia, Chile, and Thailand; and there were no gender differences in 8 of the 48 sites. For AFR, 37.7% currently smoked cigarettes in Niger; while less than 10% smoked in the other 3 AFR sites. Current cigarette smoking ranged from over 20% in Gaza Strip/West Bank (22.7%) and Lebanon (28.2%) to less than 10% (Egypt, Iran, Sudan, and Tunisia) in EMR. In EUR, current cigarette smoking was over 30% in every site, except Armenia, Czech Republic, Lithuania, and Slovenia. In AMR, current cigarette smoking was over 20% in all sites, except Brazil, Jamaica, and Panama. In the SEAR sites, current cigarette smoking was over 20% in Bangladesh and Nepal and less than 5% in Sri Lanka and Thailand. In WPR, current cigarette smoking ranged from 16.0% in South Korea to 6.4% in Cambodia.

**Table 3:** Prevalence of Current Tobacco Use, by Sex, Region and Country, Medical Global Health Professions Student Survey 2005-2008

		Current cigarette smokers				Current users of other tobacco products			
		
Country (Site)	Year	Total % (95% CI)	Male % (95% CI)	Female % (95% CI)	P-Value	Total % (95% CI)	Male % (95% CI)	Female % (95% CI)	P-Value
**AFRICAN REGION(AFR)**									

Algeria	2007	9.0 (8.1 - 9.9)	23.7 (21.3 - 26.2)	3.0 (2.5 - 3.7)	0.0000	4.1 (3.6 - 4.7)	11.0 (9.5 - 12.8)	1.1 (0.8 - 1.5)	0.0000

Kenya	2008	9.8 (7.7 - 12.5)	10.9 (8.0 - 14.8)	8.7 (5.9 - 12.6)	0.3497	4.9 (3.5 - 7.0)	5.1 (3.2 - 8.1)	4.9 (2.9 - 8.1)	0.8937

Niger	2008	37.7 (33.9 - 41.7)	42.2 (37.9 - 46.7)	15.0 (9.6 - 22.8)	0.0000	27.7 (22.0 - 34.3)	41.9 (31.4 - 53.3)	18.6 (12.6 - 26.8)	0.0006

Uganda	2005	2.8 (1.8 - 4.2)	4.1 (2.7 - 6.3)	0.0	0.0000	0.7 (0.3 - 1.8)	1.1 (0.5 - 2.6)	0.0	0.0225

**EASTERN MEDITERRANEAN REGION (EMR)**									

Egypt	2005	7.9 (5.7 - 10.7)	12.9 (9.9 - 16.5)	1.2 (0.5 - 3.0)	0.0000	7.4 (5.4 - 9.9)	11.9 (9.2 - 15.1)	1.4 (0.9 - 2.3)	0.0000

Gaza Strip/West Bank	2007	22.7 (19.7 - 26.0)	39.1 (33.8 - 44.6)	9.4 (6.8 - 12.8)	0.0000	12.3 (10.0 - 14.9)	24.1 (19.7 - 29.0)	2.4 (1.2 - 4.6)	0.0000

Iran	2007	5.6 (4.6 - 6.9)	11.6 (9.0 - 14.9)	2.8 (1.9 - 4.0)	0.0000	9.9 (8.5 - 11.6)	16.2 (13.0 - 19.9)	7.0 (5.5 - 8.8)	0.0000

Iraq	2005	17.5 (15.4 - 19.8)	20.4 (17.2 - 24.1)	15.1 (12.5 - 18.2)	0.0201	7.8 (6.4 - 9.5)	4.7 (3.2 - 6.9)	10.3 (8.1 - 13.0)	0.0003

Lebanon	2006	28.2 (25.1 - 31.4)	36.1 (31.8 - 40.7)	18.0 (14.4 - 22.3)	0.0000	21.8 (19.2 - 24.8)	21.7 (18.2 - 25.8)	21.9 (18.1 - 26.4)	0.9449

Libyan Arab Jamahiriya	2006	10.1 (9.1 - 11.2)	22.3 (20.1 - 24.6)	1.9 (1.4 - 2.7)	0.0000	8.3 (7.4 - 9.3)	15.3 (13.6 - 17.3)	3.6 (2.8 - 4.5)	0.0000

Saudi Arabia	2006	11.6 (9.2 - 14.6)	13.1 (10.0 - 16.9)	9.6 (6.2 - 14.8)	0.2156	12.8 (10.2 - 15.9)	13.9 (10.7 - 17.7)	11.3 (7.3 - 17.0)	0.3900

Sudan	2007	7.7 (6.3 - 9.4)	19.6 (15.9 - 23.9)	1.3 (0.8 - 2.3)	0.0000	3.1 (2.2 - 4.2)	8.0 (5.8 - 10.9)	0.4 (0.2 - 1.1)	0.0000

Syrian Arab Republic	2006	16.8 (16.2 - 17.5)	24.3 (23.3 - 25.3)	4.8 (4.2 - 5.5)	0.0000	23.6 (22.8 - 24.4)	30.6 (29.6 - 31.7)	12.1 (11.2 - 13.2)	0.0000

Tunisia	2007	9.9 (8.6 - 11.3)	26.5 (22.7 - 30.7)	4.1 (3.2 - 5.4)	0.0000	7.4 (6.3 - 8.7)	20.6 (17.2 - 24.5)	3.0 (2.2 - 4.1)	0.0000

**EUROPEAN REGION (EUR)**									

Albania	2005	43.3 (40.7 - 45.9)	65.1 (59.8 - 69.9)	35.7 (32.8 - 38.7)	0.0043	1.5 (1.0 - 2.2)	3.1 (1.8 - 5.4)	1.0 (0.6 - 1.8)	0.3508

Armenia	2006	20.4 (16.1 - 25.4)	52.5 (41.7 - 63.0)	8.3 (5.2 - 13.1)	0.0000	3.1 (1.7 - 5.6)	9.5 (4.9 - 17.7)	0.7 (0.2 - 3.2)	0.0061

Bosnia & Herzegovina	2006	40.3 (39.2 - 41.5)	45.0 (43.0 - 47.0)	37.8 (36.4 - 39.2)	0.1689	8.7 (8.1 - 9.4)	10.9 (9.7 - 12.2)	7.5 (6.8 - 8.3)	0.0417

Croatia	2005	36.6 (34.1 - 39.2)	35.9 (31.5 - 40.4)	37.1 (34.1 - 40.3)	0.6480	10.7 (9.2 - 12.4)	20.2 (16.8 - 24.2)	6.3 (5.0 - 8.0)	0.0000

Czech Republic	2006	21.7 (19.5 - 24.0)	26.2 (21.9 - 30.9)	19.8 (17.2 - 22.5)	0.0158	7.4 (6.1 - 9.0)	11.1 (8.3 - 14.6)	5.8 (4.5 - 7.6)	0.0035

Kyrgyzstan	2008	36.6 (33.9 - 39.4)	50.0 (45.5 - 54.5)	27.9 (24.8 - 31.3)	0.0000	5.4 (4.2 - 6.8)	11.4 (8.8 - 14.5)	1.5 (0.8 - 2.6)	0.0000

Lithuania	2006	27.3 (23.5 - 31.4)	48.6 (39.7 - 57.7)	19.5 (15.8 - 23.8)	0.0000	15.1 (12.3 - 18.5)	33.2 (25.4 - 42.1)	8.2 (6.0 - 11.2)	0.0000

Russian Federation	2006	38.8 (37.6 - 39.9)	46.1 (44.2 - 48.1)	34.9 (33.5 - 36.2)	0.0000	10.9 (10.2 - 11.6)	19.3 (17.8 - 20.9)	6.5 (5.8 - 7.2)	0.0000

Serbia	2006	34.7 (33.2 - 36.2)	31.2 (28.7 - 33.8)	36.7 (34.9 - 38.6)	0.0007	18.0 (16.8 - 19.2)	17.7 (15.7 - 19.8)	18.4 (17.0 - 20.0)	0.5587

Slovakia	2006	30.4 (29.0 - 31.9)	36.5 (33.6 - 39.4)	27.9 (26.2 - 29.6)	0.0000	6.4 (5.7 - 7.2)	8.6 (7.1 - 10.5)	5.6 (4.8 - 6.5)	0.0017

Slovenia	2007	20.9 (17.3 - 25.0)	22.5 (15.9 - 30.8)	20.2 (16.0 - 25.1)	0.6020	5.6 (3.8 - 8.2)	14.3 (9.2 - 21.5)	2.0 (0.9 - 4.4)	0.0002

**REGION OF THE AMERICAS (AMR)**									

Argentina	2005	35.5 (33.6 - 37.4)	33.4 (30.4 - 36.4)	36.5 (34.1 - 39.1)	0.1140	6.4 (5.4 - 7.4)	9.2 (7.5 - 11.3)	4.5 (3.5 - 5.7)	0.0000

Bolivia	2006	41.1 (35.3 - 47.2)	50.3 (43.9 - 56.7)	31.7 (24.9 - 39.4)	0.0017	10.6 (8.7 - 12.8)	14.3 (11.1 - 18.4)	6.7 (4.9 - 9.0)	0.0075

Brazil (Rio de Janeiro)	2006	16.9 (15.6 - 18.2)	19.5 (17.5 - 21.6)	14.6 (13.1 - 16.3)	0.0002	5.2 (4.5 - 6.0)	7.1 (5.9 - 8.5)	3.4 (2.7 - 4.3)	0.0000

Chile	2008	28.4 (27.1 - 29.6)	27.1 (25.5 - 28.9)	29.3 (27.5 - 31.3)	0.0878	7.6 (6.8 - 8.4)	9.3 (8.2 - 10.5)	5.5 (4.6 - 6.5)	0.0000

Costa Rica	2006	32.8	38.6	29.3	*	13.7	19.2	10.7	*

Cuba (Havana)	2008	29.5 (27.6 - 31.4)	41.1 (38.0 - 44.3)	21.2 (19.1 - 23.5)	0.0000	4.1 (3.4 - 5.0)	4.1 (3.1 - 5.5)	4.1 (3.2 - 5.3)	0.9874

Guatemala	2008	22.5 (19.8 - 25.4)	27.5 (23.4 - 32.1)	17.8 (14.6 - 21.6)	0.0007	13.5 (11.4 - 15.9)	17.4 (14.0 - 21.4)	10.3 (7.8 - 13.3)	0.0024

Jamaica	2008	6.7 (4.3 - 10.3)	12.5 (7.1 - 21.2)	4.2 (2.1 - 8.0)	0.0286	3.8 (2.1 - 6.7)	12.5 (7.1 - 21.2)	0.0	0.0005

Mexico	2006	35.3 (29.8 - 41.3)	41.3 (36.0 - 46.8)	30.8 (24.7 - 37.6)	0.0010	4.5 (3.3 - 6.3)	6.3 (4.5 - 8.8)	2.7 (2.0 - 3.8)	0.0027

Panama	2008	11.1 (9.6 - 12.9)	11.2 (8.7 - 14.1)	11.1 (9.2 - 13.5)	0.9956	2.9 (2.1 - 3.9)	3.6 (2.3 - 5.6)	1.9 (1.1 - 3.1)	0.0578

Paraguay	2008	22.3 (20.9 - 23.8)	25.3 (23.2 - 27.7)	19.9 (18.1 - 21.8)	0.0003	6.8 (6.0 - 7.7)	8.3 (7.0 - 9.8)	5.6 (4.6 - 6.8)	0.0029

Peru	2006	32.7 (28.5 - 37.3)	43.5 (36.2 - 51.1)	22.2 (16.7 - 28.8)	0.0007	6.4 (3.4 - 11.8)	9.0 (5.2 - 14.9)	3.6 (1.4 - 9.1)	0.0003

Uruguay	2008	32.3 (31.2 - 33.3)	31.3 (29.5 - 33.2)	32.4 (31.2 - 33.7)	0.3222	5.8 (5.3 - 6.3)	12.5 (11.3 - 13.9)	2.6 (2.2 - 3.0)	0.0000

**SOUTH-EAST ASIA REGION (SEAR)**									

Bangladesh	2006	27.2 (20.8 - 34.8)	46.5 (37.6 - 55.6)	4.4 (1.2 - 14.1)	0.0000	11.9 (4.3 - 28.7)	13.3 (3.7 - 38.2)	9.9 (4.6 - 20.0)	0.5038

India	2005	11.6 (8.8 - 15.2)	17.7 (14.5 - 21.3)	2.0 (0.9 - 4.2)	0.0000	5.4 (3.5 - 8.3)	7.5 (5.2 - 10.6)	2.2 (0.8 - 6.4)	0.0028

Indonesia	2006	8.6 (5.4 - 13.5)	19.8 (12.5 - 29.7)	2.3 (1.2 - 4.5)	0.0024	0.9 (0.4 - 2.2)	2.2 (0.9 - 5.0)	0.1 (0.0 - 1.1)	0.0274

Myanmar	2006	12.4 (11.7 - 13.1)	24.7 (23.5 - 26.1)	1.2 (0.9 - 1.6)	0.0000	11.0 (10.3 - 11.6)	19.8 (18.6 - 21.0)	2.7 (2.3 - 3.2)	0.0000

Nepal	2005	23.5 (9.1 - 48.5)	34.5 (13.1 - 64.7)	4.0 (1.2 - 12.5)	0.0372	14.4 (6.7 - 28.1)	19.3 (6.8 - 43.6)	5.4 (1.0 - 23.9)	0.1694

Sri Lanka	2006	4.1 (3.4 - 5.0)	8.6 (7.0 - 10.7)	1.1 (0.7 - 1.8)	0.0000	3.6 (2.9 - 4.4)	6.8 (5.4 - 8.6)	1.4 (0.9 - 2.1)	0.0000

Thailand	2006	2.1 (1.6 - 2.9)	1.0 (0.5 - 2.1)	3.0 (2.1 - 4.3)	0.0029	1.7 (1.1 - 2.5)	2.3 (1.4 - 3.9)	0.9 (0.5 - 1.7)	0.0443

**WESTERN PACIFIC REGION (WPR)**									

Cambodia	2005	6.4 (4.1 - 9.7)	9.2 (6.0 - 13.9)	0.0	0.0000	3.6 (2.0 - 6.4)	5.3 (3.0 - 9.2)	0.0	0.0007

South Korea	2006	16.0 (12.0 - 21.1)	22.5 (18.1 - 27.5)	3.5 (1.6 - 7.2)	0.0000	4.8 (3.0 - 7.4)	6.9 (4.7 - 10.0)	1.0 (0.5 - 2.1)	0.0001

Viet Nam	2007	11.2 (10.6 - 11.7)	20.8 (19.7 - 21.9)	2.6 (2.3 - 3.0)	0.0000	3.3 (3.0 - 3.6)	5.0 (4.4 - 5.6)	1.7 (1.4 - 2.0)	0.0000

* Census and 100% school, class and student response rates

Among medical students, in the 4 AFR sites, current use of other tobacco products ranged from 27.7% in Niger to less than 5% in the other 3 AFR sites (Table 3). In EMR other tobacco use was over 20% in Lebanon and Syria but less than 10% in 6 of the 10 sites. In EUR, other tobacco use ranged from 18.0% in Serbia to less than 10% in 7 of the 11 sites. In AMR, use of other tobacco products was less than 10% in all sites, except Costa Rica (13.7%), Guatemala (13.5%), and Bolivia (10.6%). Use of other tobacco products ranged from over 10% in Bangladesh, Myanmar, and Nepal to less than 10% in the other 4 SEAR sites. In WPR, use of other tobacco products was less than 5% in all 3 sites. Males were more likely than females to use other tobacco products in 38 of 48 sites; females had a higher rate than males in Iraq; and there was no gender difference in 9 sites.

Data presented in Table 2 compared GHPSS medical current cigarette smoking prevalence with adult cigarette prevalence data compiled from the WHO Report on the Global Tobacco Epidemic (GTCR), 2008 (WHO, 2008). Of the 48 sites included that conducted the medical GHPSS, 41 sites reported adult cigarette prevalence data in the GTCR 2008 report. Among males there was no difference in cigarette prevalence in 20 sites, while in 18 sites the adult prevalence was higher than the GHPSS medical student data, and in three of the sites medical students had higher cigarette smoking rates compared to the adult population. For females, 17 sites reported no difference between the medical students and adult cigarette smoking prevalence rates, while in four sites the adult cigarettes smoking prevalence was higher than medical students, and in 19 sites the medical students had higher rates of cigarettes smoking prevalence compared to the adult population.

### Desire for Cessation

Over 60% of the students who reported that they were current cigarette smokers expressed a desire to quit smoking cigarettes in 26 of the 48 sites (Table 4). In 4 of the 48 sites less than half of the student who reported that they were current cigarette smokers expressed a desire to quit smoking cigarettes - only a quarter of the students in Bangladesh reported a desire to quit smoking cigarettes. Desire for current cigarettes smokers to quit smoking was over 55% in all of AFR sites, in 7 of the 10 sites in EMR, in 9 of 11 sites in EUR, in 10 of 13 sites in AMR, in 2 of 3 sites in WPR, and in 6 of the 7 sites in the SEAR region.

**Table 4:** Exposure to Secondhand Smoke (At Home and in Public Places) and School Policy and Enforcement Regarding Bans on Smoking, by Region and Country, Medical Global Health Professions Student Survey, 2005-2008

		In the past 7 days, had someone smoke in their presence and their home	In the past 7 days, had someone smoke in their presence other than in their home	Had an official policy banning smoking in school buildings and clinics	Had an official policy banning smoking in school buildings and clinics and the policy was enforced	Current cigarette smokers who want to stop smoking cigarettes now
		
Country (Site)	Year	Total % (95% CI)	Total % (95% CI)	Total % (95% CI)	Total % (95% CI)	Total % (95% CI)
**AFRICAN REGION (AFR)**						

Algeria	2007	27.0 (25.7 - 28.3)	38.4 (37.0 - 39.8)	29.8 (28.5 - 31.1)	25.0 (22.7 - 27.4)	68.7 (63.1 - 73.7)

Kenya	2008	43.9 (40.1 - 47.9)	64.5 (60.6 - 68.1)	60.1 (55.7 - 64.3)	44.4 (37.4 - 51.6)	58.3 (43.8 - 71.5)

Niger	2008	31.7 (30.1 - 33.3)	71.2 (69.6 - 72.7)	44.6 (42.9 - 46.3)	42.8 (40.3 - 45.3)	93.6 (89.4 - 96.3)

Uganda	2005	38.1 (34.7 - 41.5)	61.1 (57.6 - 64.5)	22.3 (18.9 - 26.2)	40.5 (31.9 - 49.8)	*

**EASTERN MEDITERRANEAN REGION (EMR)**						

Egypt	2005	45.6 (41.7 - 49.5)	78.4 (76.1 - 80.6)	31.1 (26.2 - 36.5)	41.1 (32.1 - 50.8)	66.0 (47.0 - 81.0)

Gaza Strip/West Bank	2007	63.3 (59.7 - 66.8)	67.1 (63.6 - 70.5)	30.0 (26.7 - 33.5)	18.8 (13.8 - 25.1)	*

Iran	2007	31.2 (28.8 - 33.7)	54.1 (51.5 - 56.7)	33.5 (31.1 - 36.1)	66.9 (62.5 - 71.0)	33.5 (21.7 - 47.9)

Iraq	2005	50.2 (47.4 - 53.1)	60.6 (57.8 - 63.4)	9.4 (7.9 - 11.2)	30.5 (22.1 - 40.6)	70.2 (56.1 - 81.4)

Lebanon	2006	54.7 (51.3 - 58.1)	80.7 (77.9 - 83.3)	51.6 (48.2 - 55.0)	61.9 (57.2 - 66.4)	40.4 (32.1 - 49.3)

Libyan Arab Jamahiriya	2006	45.6 (43.9 - 47.2)	59.6 (58.0 - 61.2)	11.5 (10.5 - 12.6)	44.2 (38.8 - 49.7)	72.2 (65.6 - 78.0)

Saudi Arabia	2006	31.0 (27.3 - 34.9)	60.9 (57.0 - 64.7)	59.8 (55.8 - 63.7)	75.7 (70.4 - 80.3)	75.9 (61.9 - 86.0)

Sudan	2007	40.6 (37.7 - 43.5)	68.5 (65.6 - 71.2)	18.3 (16.2 - 20.7)	68.3 (61.6 - 74.4)	92.1 (80.7 - 97.0)

Syrian Arab Republic	2006	59.9 (59.0 - 60.8)	79.3 (78.5 - 80.0)	10.2 (9.7 - 10.8)	48.6 (45.8 - 51.5)	58.8 (56.2 - 61.4)

Tunisia	2007	44.5 (42.1 - 47.0)	65.2 (62.9 - 67.5)	36.6 (34.3 - 39.1)	35.4 (31.5 - 39.5)	61.8 (51.9 - 70.8)

**EUROPEAN REGION (EUR)**						

Albania	2005	72.5 (70.3 - 74.7)	95.8 (94.7 - 96.7)	14.1 (12.1 - 16.4)	41.4 (33.7 - 49.6)	90.1 (85.7 - 93.3)

Armenia	2006	65.1 (59.5 - 70.4)	80.0 (74.9 - 84.2)	54.0 (48.1 - 59.8)	90.5 (84.8 - 94.2)	71.3 (50.2 - 86.0)

Bosnia & Herzegovina	2006	53.0 (51.8 - 54.2)	91.1 (90.4 - 91.8)	58.8 (57.6 - 59.9)	30.1 (28.7 - 31.6)	46.6 (44.4 - 48.9)

Croatia	2005	50.4 (47.9 - 53.0)	95.2 (93.9 - 96.2)	83.1 (80.7 - 85.2)	73.8 (70.8 - 76.7)	55.2 (50.0 - 60.3)

Czech Republic	2006	25.9 (23.6 - 28.4)	84.9 (82.8 - 86.7)	95.7 (94.4 - 96.7)	51.9 (49.0 - 54.8)	55.2 (47.7 - 62.6)

Kyrgyzstan	2008	41.1 (38.3 - 43.9)	82.1 (79.9 - 84.2)	50.5 (47.6 - 53.3)	70.2 (66.1 - 74.0)	84.0 (79.1 - 87.9)

Lithuania	2006	34.9 (30.7 - 39.3)	68.1 (63.9 - 72.1)	78.5 (74.3 - 82.2)	57.3 (52.3 - 62.2)	60.9 (49.2 - 71.4)

Russian Federation	2006	48.8 (47.7 - 49.9)	85.4 (84.6 - 86.2)	74.6 (73.6 - 75.6)	34.2 (33.0 - 35.5)	68.2 (66.2 - 70.2)

Serbia	2006	67.8 (66.3 - 69.2)	89.5 (88.4 - 90.4)	67.4 (65.9 - 68.9)	81.2 (79.6 - 82.6)	57.7 (54.7 - 60.8)

Slovakia	2006	50.1 (48.5 - 51.8)	79.4 (78.0 - 80.6)	85.2 (84.0 - 86.3)	48.2 (46.4 - 50.0)	59.6 (55.9 - 63.2)

Slovenia	2007	28.2 (24.1 - 32.6)	76.8 (72.5 - 80.5)	90.1 (86.9 - 92.6)	97.6 (95.5 - 98.7)	53.3 (39.0 - 67.1)

**REGION OF THE AMERICAS (AMR)**						

Argentina	2005	65.4 (63.5 - 67.2)	91.3 (90.1 - 92.3)	69.0 (66.8 - 71.1)	78.8 (76.3 - 81.1)	62.2 (58.5 - 65.7)

Bolivia	2006	46.8 (41.6 - 52.1)	74.2 (69.2 - 78.6)	34.4 (21.1 - 50.7)	61.4 (44.8 - 75.8)	65.7 (60.8 - 70.3)

Brazil (Rio de Janeiro)	2006	24.3 (22.8 - 25.7)	75.5 (74.0 - 76.9)	8.8 (7.5 - 10.2)	5.6 (3.0 - 10.5)	49.1 (43.3 - 54.8)

Chile	2008	42.2 (40.8 - 43.7)	83.2 (82.1 - 84.3)	67.5 (66.0 - 69.1)	57.6 (55.3 - 59.8)	57.7 (54.7 - 60.7)

Costa Rica	2006	45.9	87.7	69.9	75.3	65.6

Cuba (Havana)	2008	71.3 (69.4 - 73.2)	91.8 (90.7 - 92.9)	60.8 (58.7 - 62.8)	31.4 (29.0 - 33.9)	58.2 (54.1 - 62.1)

Guatemala	2008	34.6 (31.5 - 37.8)	75.0 (72.0 - 77.7)	72.7 (69.7 - 75.6)	19.4 (16.4 - 22.8)	67.7 (58.4 - 75.9)

Jamaica	2008	29.0 (24.1 - 34.4)	58.9 (53.2 - 64.4)	82.1 (77.2 - 86.1)	50.6 (44.1 - 57.0)	*

Mexico	2006	46.8 (40.4 - 53.2)	83.7 (79.3 - 87.3)	52.3 (42.3 - 62.1)	62.1 (51.3 - 71.8)	58.4 (52.0 - 64.5)

Panama	2008	29.7 (27.4 - 32.2)	51.1 (48.5 - 53.7)	75.3 (72.9 - 77.5)	66.1 (63.2 - 68.9)	69.4 (60.2 - 77.3)

Paraguay	2008	46.4 (44.7 - 48.2)	82.5 (81.1 - 83.7)	26.1 (24.6 - 27.7)	44.7 (41.3 - 48.1)	86.0 (82.3 - 89.1)

Peru	2006	37.1 (31.0 - 43.6)	65.4 (60.4 - 70.2)	31.2 (20.7 - 44.1)	59.0 (48.9 - 68.3)	59.7 (52.7 - 66.3)

Uruguay	2008	39.7 (38.6 - 40.7)	49.1 (48.0 - 50.2)	90.7 (90.0 - 91.3)	91.3 (90.6 - 91.9)	53.7 (51.4 - 56.0)

**SOUTH-EAST ASIA REGION (SEAR)**						

Bangladesh	2006	44.7 (24.9 - 66.3)	77.1 (45.9 - 93.1)	67.2 (40.7 - 86.0)	79.1 (51.8 - 93.0)	26.6 (12.4 - 48.1)

India	2005	42.8 (37.9 - 47.9)	73.7 (68.9 - 78.0)	48.0 (44.5 - 51.5)	62.8 (54.7 - 70.3)	71.8 (52.7 - 85.4)

Indonesia	2006	44.6 (37.9 - 51.4)	80.3 (72.3 - 86.3)	41.1 (24.7 - 59.8)	41.0 (29.5 - 53.6)	76.6 (53.1 - 90.4)

Myanmar	2006	54.4 (53.4 - 55.4)	78.5 (77.6 - 79.3)	68.1 (67.2 - 69.1)	52.8 (51.5 - 54.1)	74.8 (71.3 - 78.0)

Nepal	2005	45.9 (26.1 - 67.0)	69.3 (48.3 - 84.5)	53.1 (47.2 - 59.0)	56.8 (33.3 - 77.5)	65.3 (32.0 - 88.3)

Sri Lanka	2006	21.1 (19.6 - 22.7)	54.5 (52.5 - 56.4)	39.4 (37.4 - 41.4)	74.2 (70.9 - 77.3)	66.7 (55.2 - 76.5)

Thailand	2006	27.3 (25.0 - 29.7)	56.1 (53.5 - 58.6)	48.4 (45.8 - 51.0)	80.0 (76.5 - 83.1)	69.5 (46.1 - 85.8)

**WESTERN PACIFIC REGION (WPR)**						

Cambodia	2005	57.7 (52.1 - 63.0)	64.0 (58.5 - 69.1)	63.1 (57.5 - 68.3)	73.1 (65.3 - 79.7)	*

South Korea	2006	16.8 (14.4 - 19.5)	75.2 (69.5 - 80.1)	78.6 (72.5 - 83.7)	83.4 (79.0 - 87.0)	65.5 (53.6 - 75.7)

Viet Nam	2007	61.4 (60.6 - 62.3)	71.2 (70.4 - 72.0)	71.8 (71.0 - 72.6)	88.2 (87.3 - 89.1)	71.1 (68.0 - 74.1)

* Cell size less than 10

### Exposure to Secondhand Smoke (SHS)

Over 50% of the students reported that they had been exposed to SHS in their home in the past 7 days in 15 of the 48 sites (Table 4). Over 70% reported exposure to SHS at home in Albania and Cuba. Less than 50% of the students in all 4 AFR sites reported exposure to SHS at home in the past 7 days; whereas exposure at home was greater than 50% in 4 of 10 sites in EMR, in 6 of 11 sites in EUR, in 2 of 13 sites in AMR, in 2 of 3 sites in WPR and in Myanmar in SEAR sites.

Over 70% of the students reported that they had been exposed to SHS in public places in the past 7 days in 29 of the 48 sites (Table 4). Exposure to SHS in public places was greater than 60% in 3 of 4 sites in AFR and in all 3 WPR sites; greater than 70% in 3 of 10 sites in EMR and in 9 of 13 sites in AMR and greater than 80% in 8 of 11 sites in EUR (Table 4).

The proportion of students reporting their schools have an official policy banning smoking in school buildings and clinics was over 60% in 21 of the 48 sites; and less than 20% in 6 sites (Table 4). Having a policy was least likely in EMR and most likely in EUR and WPR. Over 70% of the students reported enforcement of the policy in 15 of the 48 sites. Enforcement was less than 30% in Algeria, Gaza Strip/West Bank, Brazil, and Guatemala.

### Health Professional Roles and Training

Over 80% of the students thought health professionals have a role in giving advice about smoking cessation to patients in 42 of 46 sites, with 30 over 90% (Table 5). The lowest was in Slovakia (59.7%). Over 80% of the students thought health professionals should get specific training on cessation techniques in 41 of the 48 sites, with 33 over 90%. The lowest was in Czech Republic (60.8%). Less than 40% of the students reported having ever received some kind of formal training in their professional school on cessation approaches to use with their patients in 46 of the 48 sites; less than 20% in 16 sites, and less than 10% in 6 sites. Over 40% of the students had received formal training in Niger (46.4%) and Myanmar (43.7%).

**Table 5:** Attitudes Toward and Training in Patient Smoking Cessation Counseling, by Region and Country, Medical Global Health Professions Student Survey, 2005-2008

		Thought health professionals have a role in giving advice or information about smoking cessation to patients	Thought health professionals should get specific training on cessation techniques	Had ever received any formal training in smoking cessation approaches to use with patients in their medical school training
		
Country (Site)	Year	Total % (95% CI)	Total % (95% CI)	Total % (95% CI)
**AFRICAN REGION (AFR)**				

Algeria	2007	85.6 (84.6 - 86.7)	89.7 (88.7 - 90.5)	36.1 (34.7 - 37.5)

Kenya	2008	98.2 (96.8 - 99.0)	96.9 (95.2 - 98.0)	26.0 (22.7 - 29.6)

Niger	2008	100.0	97.2 (96.6 - 97.8)	46.4 (44.7 - 48.1)

Uganda	2005	98.8 (97.7 - 99.3)	97.3 (95.9 - 98.2)	15.9 (13.5 - 18.6)

**EASTERN MEDITERRANEAN REGION (EMR)**				

Egypt	2005	91.1 (89.6 - 92.4)	92.5 (90.4 - 94.2)	20.9 (18.4 - 23.6)

Gaza Strip/West Bank	2007	88.4 (85.7 - 90.6)	95.1 (93.3 - 96.5)	25.6 (22.5 - 28.9)

Iran	2007	91.7 (90.1 - 93.1)	94.8 (93.5 - 95.8)	10.0 (8.4 - 11.8)

Iraq	2005	63.8 (61.0 - 66.5)	77.9 (75.4 - 80.2)	31.3 (28.7 - 34.0)

Lebanon	2006	88.9 (86.7 - 90.8)	94.0 (92.1 - 95.4)	29.7 (26.7 - 33.0)

Libyan Arab Jamahiriya	2006	77.4 (76.0 - 78.8)	87.1 (85.9 - 88.1)	24.9 (23.4 - 26.3)

Saudi Arabia	2006	97.8 (96.3 - 98.7)	89.1 (86.4 - 91.4)	6.4 (4.6 - 8.7)

Sudan	2007	97.9 (96.8 - 98.6)	97.5 (96.4 - 98.3)	31.6 (28.9 - 34.5)

Syrian Arab Republic	2006	95.7 (95.3 - 96.0)	96.8 (96.5 - 97.1)	29.4 (28.5 - 30.2)

Tunisia	2007	96.5 (95.5 - 97.3)	94.0 (92.8 - 95.1)	23.0 (20.9 - 25.1)

**EUROPEAN REGION (EUR)**				

Albania	2005	95.0 (93.8 - 95.9)	97.1 (96.2 - 97.8)	10.3 (9.0 - 11.9)

Armenia	2006	80.8 (75.8 - 85.0)	84.4 (79.7 - 88.2)	34.1 (28.9 - 39.8)

Bosnia & Herzegovina	2006	86.0 (85.1 - 86.8)	86.4 (85.5 - 87.1)	11.2 (10.5 - 12.0)

Croatia	2005	97.7 (96.8 - 98.4)	71.7 (69.3 - 73.9)	14.5 (12.8 - 16.4)

Czech Republic	2006	82.0 (79.8 - 84.0)	60.8 (58.1 - 63.5)	1.4 (0.9 - 2.2)

Kyrgyzstan	2008	85.7 (83.6 - 87.6)	92.0 (90.3 - 93.4)	22.3 (20.1 - 24.8)

Lithuania	2006	87.6 (84.3 - 90.2)	96.1 (94.1 - 97.4)	25.2 (21.6 - 29.2)

Russian Federation	2006	NA	77.1 (76.2 - 78.1)	20.6 (19.7 - 21.6)

Serbia	2006	89.9 (88.9 - 90.8)	81.5 (80.2 - 82.7)	21.3 (20.0 - 22.6)

Slovakia	2006	59.7 (58.0 - 61.3)	71.4 (69.9 - 72.8)	3.0 (2.5 - 3.6)

Slovenia	2007	100.0	77.5 (73.3 - 81.2)	5.6 (3.8 - 8.2)

**REGION OF THE AMERICAS (AMR)**				

Argentina	2005	98.8 (98.3 - 99.1)	91.3 (90.1 - 92.3)	5.2 (4.4 - 6.1)

Bolivia	2006	87.6 (84.4 - 90.2)	95.5 (93.4 - 97.0)	20.4 (14.3 - 28.3)

Brazil (Rio de Janeiro)	2006	81.9 (80.5 - 83.1)	94.3 (93.5 - 95.1)	21.2 (19.9 - 22.7)

Chile	2008	97.5 (97.0 - 97.9)	93.4 (92.7 - 94.0)	23.4 (22.1 - 24.6)

Costa Rica	2006	97.1	94.9	11.2

Cuba (Havana)	2008	100.0	98.7 (98.2 - 99.1)	28.9 (27.1 - 30.8)

Guatemala	2008	100.0	96.3 (94.9 - 97.4)	23.6 (20.8 - 26.5)

Jamaica	2008	99.1 (97.0 - 99.7)	94.3 (91.0 - 96.5)	8.5 (5.8 - 12.3)

Mexico	2006	85.8 (83.0 - 88.3)	94.7 (93.0 - 96.0)	20.4 (14.5 - 27.8)

Panama	2008	100.0	94.6 (93.3 - 95.7)	26.1 (23.8 - 28.4)

Paraguay	2008	96.0 (95.3 - 96.6)	98.8 (98.4 - 99.1)	30.8 (29.2 - 32.4)

Peru	2006	95.9 (93.1 - 97.5)	97.5 (94.2 - 98.9)	30.0 (21.9 - 39.4)

Uruguay	2008	93.0 (92.4 - 93.6)	89.5 (88.8 - 90.2)	10.9 (10.2 - 11.6)

**SOUTH-EAST ASIA REGION (SEAR)**				

Bangladesh	2006	71.1 (45.2 - 88.0)	87.8 (81.2 - 92.3)	24.7 (12.2 - 43.6)

India	2005	96.9 (95.1 - 98.0)	91.0 (89.3 - 92.5)	22.3 (18.3 - 26.9)

Indonesia	2006	98.1 (97.1 - 98.7)	95.8 (92.9 - 97.5)	22.2 (5.3 - 59.2)

Myanmar	2006	94.8 (94.3 - 95.2)	96.4 (96.0 - 96.8)	43.7 (42.7 - 44.7)

Nepal	2005	97.0 (93.8 - 98.5)	93.0 (85.1 - 96.8)	21.9 (12.8 - 34.9)

Sri Lanka	2006	93.9 (92.9 - 94.8)	90.6 (89.4 - 91.7)	16.2 (14.8 - 17.7)

Thailand	2006	95.7 (94.5 - 96.6)	93.1 (91.7 - 94.3)	24.0 (21.8 - 26.3)

**WESTERN PACIFIC REGION (WPR)**				

Cambodia	2005	99.1 (97.1 - 99.7)	99.1 (97.1 - 99.7)	14.4 (10.9 - 18.8)

South Korea	2006	93.3 (89.0 - 96.0)	76.6 (72.6 - 80.2)	18.9 (14.6 - 24.2)

Viet Nam	2007	NA	92.0 (91.5 - 92.5)	27.2 (26.4 - 28.0)

NA Data not available

## Discussion

### Tobacco Use

Findings from the Medical GHPSS show that over 20% of medical students currently smoke cigarettes in 26 of 48 sites and over 40% in 3 sites. Use of other forms of tobacco was over 10% in 15 of 48 sites and over 20% in 2 sites. Not only does tobacco use endanger the health of medical students, but it is also known to negatively influence the health professionals to deliver effective anti-tobacco counseling when they start seeing patients [13]. Physicians who have healthy personal habits are more likely to discuss related preventive behaviors with their patients [14]. Thus the current smoking prevalence rates for medical students are disconcerting given that among the 41 sites that contained adult smoking prevalence data; over 55% reported no difference between medical students and the general adult population or the medical students had higher rates. For males, in 23 of the 41 (56.1%) sites there was no difference or male medical students had higher rates of smoking compared to the general adult male population. While among females, female medical students had higher rates of cigarette smoking prevalence compared to the general adult female population in 36 of the 40 (90%) countries, or there was no difference.

Since physician counseling on smoking cessation can have a positive effect on patient's smoking habits health promotion programs including tobacco cessation services for medical students may play a significant role in the future delivery of patient cessation counseling practices [15]. Therefore educational institutions training medical students should help their students quit using tobacco by providing encouragement and information to students who are considering quitting and providing assistance to students who are motivated to quit.

### Exposure to Secondhand Smoke (SHS)

Medical schools/colleges should be encouraged to provide smoke-free work and study areas by banning smoking in their buildings and clinics. A smoke-free work environment has been shown to improve air quality, reduce health problems associated with exposure to tobacco smoke, support and encourage cessation attempts among smokers trying to quit, and receive high levels of public support from people who spend time in the area [16]. Furthermore, the creation of smoke-free areas by educational institutions sends a clear message to educators, students, patients, and clinicians about the negative impact of tobacco [17]. Results from the Medical GHPSS show high exposure to SHS: over 50% of the medical students reported they were exposed to SHS in their home in 15 of the 48 sites and over 70% were exposed to SHS in public places in 29 of the 48 sites.

### Health Professional Training

Medical students should be trained to provide effective, accurate, and accessible advice to patients on all aspects of health. Results from the Medical GHPSS show that over 80% of medical students recognize that they are role models in society; over 80% think they should receive training on counseling and treating patients to quit using tobacco, but less than 40% have received formal training.

The Medical GHPSS surveyed 3^rd ^year students, so it is possible that students receive training on patient cessation techniques during the latter years of their programs. To address this possibility, the GHPSS research coordinators raised this question to the school administrators and found that, in the majority of the countries, there is no formal training at any time. Of the countries with some training, the type of training included: problem-based learning, included in general counseling curricula; or were included in curricula as part of community medicine or public health courses. This study did not make an effort to evaluate the adequacy of cessation training in the countries reporting this type of instruction. However, professional training for medical students should include courses detailing the harmful health effects of tobacco use and exposure to SHS, and training in counseling on tobacco cessation techniques [1].

### Limitations

The Medical GHPSS is subject to at least four limitations. First, this study reflects 3^rd ^year students who have not had substantial interaction with patients, so these survey results should not be extrapolated to account for practicing health professionals. Second, the sites including in this study are not representative of individual WHO regions given the number of sites included per region **(**of the 193 WHO Member States we report data for 47 sites and one geographic region). Third, since the adult smoking rates cited from the WHO Report on the Global Tobacco Epidemic (GTCR) 2008, were not collected using a standardized and consistent methodology the comparison of current cigarette smoking prevalence rates should be interpreted with caution. Lastly, data were based on the self-report of students, who might underreport or over-report their behaviors or attitudes. The extent of this bias cannot be determined from these data; however, reliability studies in the United States have indicated good test-retest results for similar tobacco-related questions [18].

## Conclusions

Educational institutions, public health organizations, and education officials should discourage tobacco use among medical students and all health professionals and work together to design and implement programs that train medical students in effective cessation-counseling techniques by utilizing evidence-based strategies. Previous studies have noted the following problems in preparing medical students for a role in tobacco control: 1) a lack of tobacco-related material in medical school curricula [19-22]; and 2) practicing physicians report difficulties delivering tobacco cessation care to patients due to lack of time, reluctance to get involved in personal issues, and failure to use evidence-based methods with patients [23]. Despite these barriers, structural and institutional interventions such as creating tobacco-free hospitals and providing insurance coverage for cessation services combined with practice-level strategies such as including tobacco cessation training in expected physician competency measures could foster a positive environment for tobacco control in the healthcare delivery system. This combined approach among health institutions and medical professionals should improve the incorporation of tobacco cessation into standard healthcare delivery [24].

The Medical GHPSS has shown global gaps in medical school training to provide effective patient tobacco cessation counseling to their future patients. Tobacco control efforts in these countries should include programmatic efforts that help train medical students to deliver these counseling services that can assist people in changing their smoking behavior. This is particularly important for the sites included in this study that have ratified the WHO FCTC (43 of the 47 sites) who are legally obligated to implement the provisions of the Articles under the treaty. This study also shows strong interest on the part of the students in receiving this training, despite previous studies that suggest a lack of interest among students to learn about the health consequences of tobacco use [22]. The Medical GHPSS is helpful in evaluating the behavior and attitudes regarding tobacco use among medical students, but additional research is necessary to improve the evidence base for effective tobacco-related curricula, especially materials that are appropriate for a range of cultural and economic settings. Repeating the Medical GHPSS will prove useful in evaluating the effectiveness of tobacco-related curricula for future health professionals in these countries.

## Competing interests

The authors declare that they have no competing interests.

## Authors' contributions

CWW, JL, VL and NRJ conceived of the study, participated in its design and coordination, and helped to draft the manuscript. DNS participated in interpretation of the statistical analysis and drafting of the manuscript. All authors read and approved the final manuscript.

## Other Notes

CW Warren, J Lee and V Lea are obligated by their institution to have the following statement printed in the report: "The findings and conclusions in this report are those of the authors and do not necessarily represent the views of the Centers for Disease Control and Prevention."

## Pre-publication history

The pre-publication history for this paper can be accessed here:

http://www.biomedcentral.com/1471-2458/11/72/prepub
